# Supplementary material for: Resolving the full spectrum of human genome variation using Linked-Reads
Source: Genome Res. 2019 Apr;29(4):635–45. doi: 10.1101/gr.234443.118 (PMC6442396; doi:10.1101/gr.234443.118)
Supplement: Supplemental Material [file supp_gr.234443.118_Supplemental_longranger-2.2.zip › longranger-2.2.tar.bz2!/lr2.2-public/lib/stan/cmdstan/stan/doc/api/html/transpose_8hpp_source.html]

Stan: src/stan/math/matrix/transpose.hpp Source File


|  |  |
| --- | --- |
|  | Stan  2.5.0  probability, sampling & optimization |


- Main Page
- Related Pages
- Namespaces
- Classes
- Files

- File List
- File Members

All Classes Namespaces Files Functions Variables Typedefs Enumerations Enumerator Friends Macros Pages

- src
- stan
- math
- matrix

transpose.hpp

Go to the documentation of this file.

1 #ifndef STAN\_\_MATH\_\_MATRIX\_\_TRANSPOSE\_HPP

2 #define STAN\_\_MATH\_\_MATRIX\_\_TRANSPOSE\_HPP

3

4 #include <stan/math/matrix/Eigen.hpp>

5

6 namespace stan {

7  namespace math {

8

9  template <typename T, int R, int C>

10  Eigen::Matrix<T,C,R>

11  inline

12  transpose(const Eigen::Matrix<T,R,C>& m) {

13  return m.transpose();

14  }

15

16  }

17 }

18 #endif

stan::math::transpose

Eigen::Matrix< T, C, R > transpose(const Eigen::Matrix< T, R, C > &m)

**Definition:** transpose.hpp:12

Eigen.hpp


---

[ Stan Home Page ]

*© 2011–2014,
Stan Development Team.*
